# Exploratory Analysis of Glaucoma-Associated SNPs in a Colombian Cohort Highlights Potential Involvement of Oxidative, Vascular, and Neurodegenerative Pathways

**DOI:** 10.3390/genes17080919

**Published:** 2026-08-04

**Authors:** Carlos Casanova, Claudia Valencia-Peña, Wilmar Saldarriaga-Gil, Edgar Lozano-Cruz, Andrés Castillo

**Affiliations:** 1 TAOLab-OneHealth, Faculty of Natural & Exact Sciences, Universidad del Valle, Cali 760032, Colombia; casanova.carlos@correounivalle.edu.co; 2 Faculty of Health, Universidad del Valle, Cali 760043, Colombia; claudia.valencia.pena@correounivalle.edu.co (C.V.-P.); wilmar.saldarriaga@correounivalle.edu.co (W.S.-G.); edgar.lozano@correounivalle.edu.co (E.L.-C.); 3 Hospital Universitario del Valle, Cali 760042, Colombia; 4 Clínica de Oftalmología de Cali, Cali 760036, Colombia; 5 Clínica Vision IA, Cali 760046, Colombia

**Keywords:** glaucoma, SNPs, oxidative stress, extracellular matrix, neurodegeneration, vascular dysfunction, genetics

## Abstract

Background/Objectives: Primary open-angle glaucoma (POAG) is a complex multifactorial optic neuropathy involving genetic, vascular, oxidative, inflammatory, and neurodegenerative mechanisms. Despite advances in genome-wide studies, the contribution of genetic variants remains incompletely characterized in underrepresented Latin American populations. This study aimed to characterize the genetic landscape of POAG in a Colombian cohort by identifying previously reported glaucoma-associated variants, rare candidate variants, and pharmacogenomic markers and integrating these findings into biologically relevant pathways. Methods: An exploratory descriptive study was conducted in 21 Colombian patients with confirmed POAG. Whole-exome sequencing (WES) was performed at an average sequencing depth of approximately 100×. Variants were quality-filtered, functionally annotated, and prioritized within 446 POAG-associated genes retrieved from DisGeNET. Previously reported glaucoma-associated variants and rare candidate variants were identified, while pharmacogenomic variants related to latanoprost and timolol response were evaluated using ClinPGx/PharmGKB. Identified genes were classified according to major biological pathways relevant to glaucoma pathophysiology. Results: Of the 446 POAG-associated genes, 381 were detected in the patients’ exomes. A total of 10,220 molecular variants were identified, of which 1,187 synonymous variants were excluded, leaving 9,033 variants for downstream analysis. Among these, 955 were non-synonymous SNVs, including 26 variants previously reported in association with glaucoma and 929 potentially novel coding variants. Previously reported variants included loci in SIX6, LOXL1, CYP1B1, NOS3, and SOD2. Two rare candidate variants (minor allele frequency <1%) were identified in FMNL2 and C3. Pharmacogenomic variants in PTGS1, ADRB1, and ABCC4 with potential implications for response to latanoprost or timolol were also detected. Functional integration highlighted pathways involving oxidative stress, extracellular matrix remodeling, vascular regulation, neurodegeneration, and inflammation. Conclusions: This exploratory analysis identifies known glaucoma-associated variants, rare candidate variants, and pharmacogenomic markers in Colombian patients with POAG. The findings support a multifactorial biological framework involving interconnected oxidative, structural, vascular, neurodegenerative, and inflammatory pathways. The FMNL2 and C3 variants represent candidates for further investigation, while the identified pharmacogenomic variants highlight the potential relevance of genomic profiling for personalized glaucoma management. Larger ancestry-informed case–control studies are required to validate these observations and determine their clinical significance.

## 1. Introduction

Glaucoma is a progressive optic neuropathy characterized by the degeneration of retinal ganglion cells (RGCs) and irreversible visual field loss, representing one of the leading causes of blindness worldwide [1,2]. Among its clinical subtypes, primary open-angle glaucoma (POAG) is the most prevalent, with a rapidly increasing global burden projected to affect over 110 million individuals by 2040 [2]. The disease disproportionately affects populations of African and Hispanic ancestry, highlighting the importance of studying its genetic basis in underrepresented populations [1,3].

Traditionally, glaucoma has been associated with elevated intraocular pressure (IOP), which remains the main modifiable risk factor. However, growing evidence indicates that glaucoma is a complex and multifactorial disease involving genetic predisposition, oxidative stress, vascular dysregulation, and neurodegeneration [4,5]. This change in basic assumptions has led to the recognition of glaucoma as a neurodegenerative disorder with systemic components, rather than a condition solely driven by mechanical pressure.

From a genetic perspective, large-scale genome-wide association studies (GWAS) have identified multiple susceptibility loci, including *SIX6*, *CDKN2B-AS1*, and *LOXL1*, which participate in ocular development, cell cycle regulation, and extracellular matrix remodeling [6,7]. Despite these advances, the functional integration of genetic variants into biological pathways remains incompletely understood. Moreover, most genomic studies have been conducted in European or Asian populations, limiting the extrapolation of findings to genetically admixed populations such as those in Latin America [8].

In Colombia, the prevalence of POAG has been estimated at 5.6% among individuals over 40 years of age with comorbid conditions such as hypertension and diabetes [3]. This high prevalence, combined with the country’s genetic diversity, underscores the need to investigate population-specific genetic factors contributing to disease susceptibility and progression [8]. Furthermore, interethnic variation in genetic background has been shown to influence both disease risk and pharmacological response, adding complexity to disease management [9].

In addition to disease susceptibility, variability in treatment response represents a major clinical challenge. First-line therapies, including prostaglandin analogs such as latanoprost and beta-blockers such as timolol, effectively reduce IOP; however, substantial interindividual variability in therapeutic response has been reported [10,11]. Pharmacogenomic studies suggest that genetic variation in drug metabolism, transport, and receptor signaling pathways may contribute to these differences, although evidence in glaucoma remains limited [11,12].

Whole-exome sequencing (WES) has emerged as a powerful approach for identifying coding variants and uncovering rare, potentially high-impact genetic alterations in complex diseases [7]. Unlike GWAS, which primarily detects common variants, WES enables a more comprehensive exploration of functional genetic variation. However, exome-based studies in glaucoma remain scarce, particularly in Latin American populations.

Several hypotheses have been proposed to explain glaucoma pathogenesis. The mechanical theory emphasizes elevated IOP and structural damage to the optic nerve, whereas the vascular theory highlights impaired ocular blood flow and ischemia [5]. More recently, integrative models incorporating oxidative stress, mitochondrial dysfunction, and neuroinflammation have gained support, suggesting that multiple biological processes interact synergistically to drive disease progression [5,13].

Understanding the genetic architecture of glaucoma requires integrating genomic, functional, and pharmacogenomic data. Therefore, the aim of this study was to characterize the genetic landscape of POAG in patients from southwestern Colombia using whole-exome sequencing. Specifically, we sought to identify previously reported glaucoma-associated variants, detect rare candidate variants, and evaluate pharmacogenomic markers, as well as to integrate these findings into biologically relevant pathways.

Collectively, this study provides novel insights into the genetic architecture of glaucoma in an underrepresented population and supports a multifactorial model of disease involving oxidative stress, extracellular matrix remodeling, vascular dysfunction, neurodegeneration, and inflammation.

## 2. Materials and Methods

### 2.1. Study Design, Population, and Sample Collection

An exploratory descriptive study was conducted to identify molecular variants associated with POAG. Because this investigation was designed as an exploratory descriptive study without an unaffected control group, no inferential statistical analyses were performed. The primary objective was to identify glaucoma-associated molecular variants and generate hypotheses for future studies rather than to evaluate statistical associations between cases and controls. Therefore, results are presented descriptively using variant counts, frequencies, and functional annotations. All participants were Colombian nationals residing in Colombia and were recruited from ophthalmology referral centers in southwestern Colombia. Nationality was established from the clinical records and self-reported information collected during enrollment. Because ancestry-informative genetic markers were not evaluated, no inference regarding genetic ancestry was performed, and the term “Colombian” refers to nationality rather than genetic ancestry. A total of twenty-one patients with a confirmed diagnosis of POAG in one or both eyes were included after providing written informed consent and meeting the eligibility criteria. Inclusion criteria comprised: (i) self-reported Colombian nationality and birth in Colombia; (ii) age ≥ 18 years; (iii) confirmed diagnosis of primary open-angle glaucoma (POAG); and (iv) ongoing monotherapy treatment with either latanoprost or timolol. Exclusion criteria included cognitive or psychiatric conditions impairing informed consent, baseline IOP ≤ 21 mmHg prior to treatment, ocular comorbidities affecting accurate IOP measurement (e.g., infections or corneal irregularities), prior use of other ocular hypotensive medications, concomitant systemic beta-blocker therapy, and history of glaucoma-related surgical interventions (including laser trabeculoplasty, trabeculectomy, valvular implants, or cyclodestructive procedures). Individuals who were not Colombian nationals or who had been born outside Colombia were excluded from the study.

The study was conducted in accordance with the Declaration of Helsinki and approved by the Institutional Ethics Committee of the Clínica de Oftalmología de Cali (approval code: 109-2020, approval date: 18 July 2020) and Hospital Universitario del Valle (approval code: 050-2020, approval date: 28 August 2020).

Peripheral blood samples were collected from all participants for genomic DNA extraction using the salting-out method. DNA concentration and purity were assessed using a Nanodrop^®^ ND-2000c spectrophotometer (Thermo Scientific^®^, Waltham, MA, USA) following standard protocols.

### 2.2. Whole-Exome Sequencing and Bioinformatic Processing

WES was performed for all samples using Illumina HiSeq 2000 technology, achieving an average sequencing depth of approximately 100×.

Raw sequencing reads underwent quality control using FastQC v0.11.7. High-quality reads were aligned to the human reference genome (GRCh38) using BWA v0.7.17 with the BWA-MEM algorithm [14]. Variant calling, including single nucleotide variants (SNVs) and INDELs, was performed using the HaplotypeCaller tool in gVCF mode from the GATK v4.0.2.1 [15].

Identified variants were filtered and functionally annotated using ANNOVAR (version 2018) [16]. Following variant calling with GATK HaplotypeCaller, quality filtering was applied to retain high-confidence variants for downstream analyses. Variants were required to pass the standard GATK quality filters (FILTER = PASS). Additional filtering criteria included a minimum sequencing depth (DP) of 10×, genotype quality (GQ) ≥ 20, and variant quality score (QUAL) ≥ 30. Variants with missing genotype calls or low-confidence genotype assignments were excluded. Multi-allelic variants were decomposed into bi-allelic records before annotation when required. High-confidence variants were subsequently annotated using ANNOVAR, incorporating gene-based functional annotation, predicted functional effects, and population allele frequencies from public databases. The resulting annotated dataset was then subjected to biological prioritization according to the strategy described in Section 2.6.

### 2.3. Identification and Functional Characterization of Glaucoma-Associated Variants

A curated list of genes associated with primary open-angle glaucoma (POAG) was retrieved from the DisGeNET knowledge platform (version 7.0, https://www.disgenet.com/), accessed on 18 December 2024 [17]. Gene retrieval was performed by querying the disease term “Primary Open-Angle Glaucoma” (POAG) using the corresponding DisGeNET disease concept (UMLS Concept Unique Identifier, CUI: C0339573; C0017612). All genes annotated as associated with POAG in the curated DisGeNET database were exported, resulting in an initial list of 446 glaucoma-associated genes. Only genes with documented evidence of association with POAG according to DisGeNET were retained for downstream analyses. This evidence integrates information from expert-curated databases, genome-wide association studies (GWAS), experimental studies, and literature mining. No additional filtering based on gene score or evidence index was applied at this stage, allowing inclusion of all genes with reported evidence of association. Subsequently, variants identified within these genes were extracted from the whole-exome sequencing dataset and subjected to the variant prioritization strategy described below. To identify novel candidate variants, additional filtering criteria were applied to variants located in genes previously associated with glaucoma. Candidate variants were selected based on their recurrence in multiple individuals within the cohort. Variant frequencies were further contrasted with publicly available population databases, including the Genome Aggregation Database (gnomAD v4.1), to contextualize their potential enrichment. Whenever available, allele frequencies across different ancestral populations (African/African American, Latino/Admixed American, European, East Asian, South Asian, and other populations represented in gnomAD) were examined, with particular attention to the rare candidate variants. This comparison was intended to determine whether the observed variants were population-specific or represented rare variants shared across multiple ancestral groups. In parallel, a curated panel of SNPs associated with glaucoma or related biological processes was compiled based on literature review and candidate gene approaches. Each variant was annotated according to gene association, variant type (missense, intronic, or regulatory), and predicted biological function.

### 2.4. Pathway-Based Analysis and Pharmacogenomic Evaluation

Annotated genes were classified into major biological pathways relevant to glaucoma pathophysiology, including oxidative stress, extracellular matrix remodeling, vascular regulation, neurodegeneration, and inflammation. This pathway-based approach enabled the identification of convergent biological mechanisms underlying the genetic architecture of the disease.

To assess potential variability in treatment response, pharmacogenomic analysis was performed using the ClinPGx/PharmGKB database (https://www.clinpgx.org/) [12], accessed on 18 May 2024. The presence of variants associated with response to latanoprost and timolol was evaluated across all samples.

### 2.5. Data Availability and Use of Artificial Intelligence

The data presented in this study are available from the corresponding author upon reasonable request. The datasets are not publicly available due to privacy and ethical restrictions.

No generative artificial intelligence tools were used for data analysis, interpretation, or generation of scientific content. AI tools were used solely for language editing and formatting purposes.

### 2.6. Variant Filtering and Prioritization Strategy

After quality filtering and functional annotation, a biological prioritization strategy was applied to identify variants with potential relevance to POAG. Given the exploratory design of this study and the absence of a control cohort, a targeted strategy was implemented to prioritize variants with potential biological significance. and the absence of a control cohort, a targeted strategy was implemented to prioritize genetic variants with potential biological relevance. The set of genes analyzed consisted of the 446 POAG-associated genes previously retrieved from the DisGeNET database as described in Section 2.3. From this curated gene set, 381 genes were identified in the exome dataset of the study participants and were retained for subsequent analyses, which integrated evidence from curated datasets, experimental studies, and literature mining.

From this initial set, genes detected in the patients’ exomes were retained for further analysis, allowing the study to focus on a predefined disease-relevant genomic space. This approach was intended to improve the interpretability of findings by anchoring the analysis in genes with prior evidence of association with glaucoma, rather than performing an unrestricted genome-wide search.

Within this framework, successive filtering steps were applied to reduce noise and prioritize potentially informative variants. Synonymous variants were excluded due to their limited predicted functional impact, and particular emphasis was placed on non-synonymous SNVs, given their higher likelihood of affecting protein function. Additional prioritization was performed by identifying variants previously reported in association with glaucoma using curated resources such as DisGeNET, while retaining remaining variants as exploratory candidates for further investigation. Only synonymous variants were excluded during the initial filtering stage because they were considered less likely to have a direct impact on protein function. No additional filtering was applied to remove intronic, untranslated region (UTR), intergenic, or other non-coding variants at this stage. Consequently, the remaining dataset of 9033 variants included both coding and non-coding molecular variants. Functional prioritization for downstream analyses focused primarily on non-synonymous SNVs occurring in glaucoma-associated genes, whereas the remaining non-coding variants were retained to provide a comprehensive overview of the molecular landscape identified within the selected gene set.

This prioritization strategy reflects a balance between hypothesis-driven and discovery-based approaches, enabling the identification of biologically supported variants while preserving the capacity to detect potentially novel or population-specific variation.

### 2.7. Statistical Analysis

No inferential statistical analyses were performed because the study included only patients diagnosed with primary open-angle glaucoma and did not include an unaffected control group. Consequently, statistical comparison tests such as Fisher’s exact test, Chi-square test, odds ratios, confidence intervals, or regression models were not appropriate. The analysis was therefore limited to descriptive statistics, including variant frequencies, functional annotation, pathway classification, and comparison with publicly available genomic databases. The findings should be considered exploratory and hypothesis-generating. Future case–control studies with larger sample sizes will enable robust statistical analyses to evaluate genetic associations.

## 3. Results

### 3.1. Demographics

Baseline demographics and clinical characteristics are summarized in Table 1. The mean age was 67 ± 12.2 years, and there were slightly more females in the study than males (52.38%). 90.5% of the patients had bilateral glaucoma and the baseline IOP was 25+/− 3.82 mmHg (for patients with bilateral disease, the eye with the higher baseline IOP was selected for analysis). Most of the patients (95%) received latanoprost monotherapy.

### 3.2. Exome Analysis and Variant Distribution

**Figure 1:**
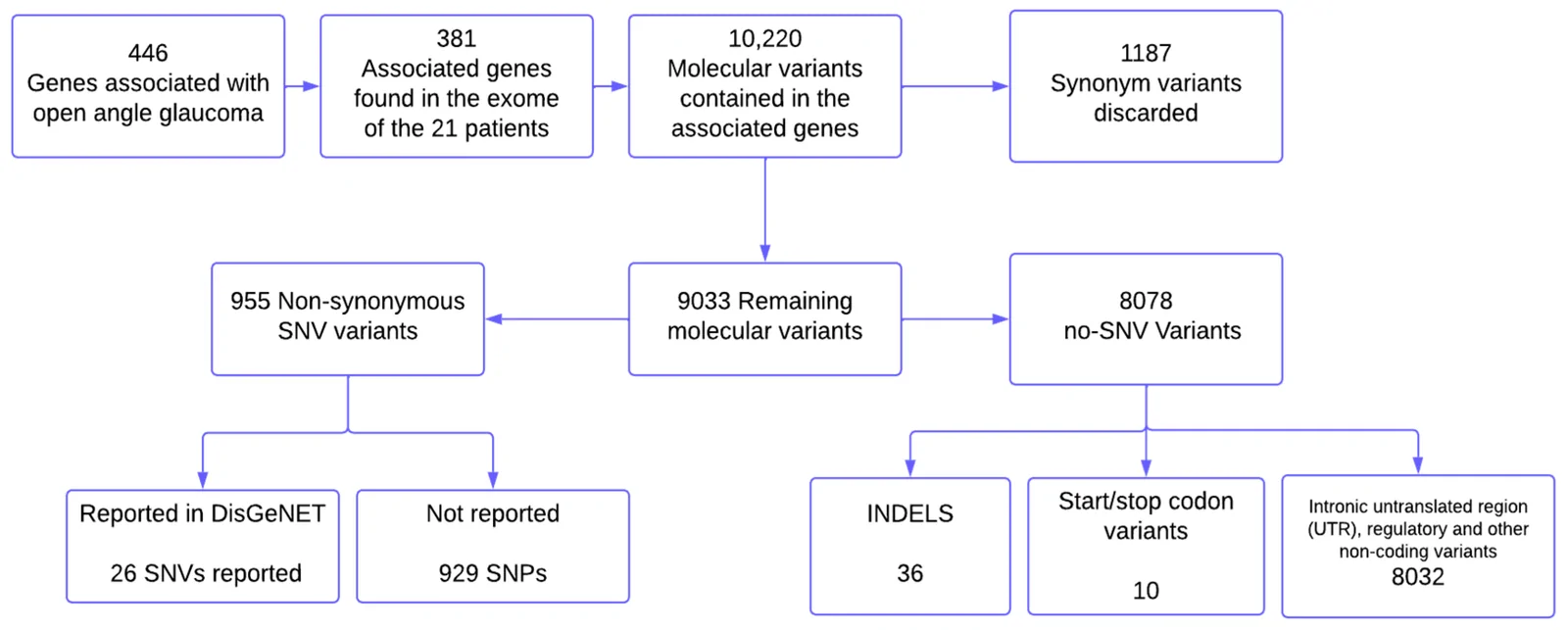
Identification and filtering of molecular variants performed in the patient cohort.

After applying the quality filtering criteria described in the Methods, high-confidence variants were retained for downstream analyses and functional prioritization. The workflow presented in Figure 1 summarizes the stepwise filtering and prioritization of genetic variants identified in the exome analysis of patients with POAG. From the initial curated set of 446 glaucoma-associated genes, 381 genes were identified in the exomes of the twenty-one patients, indicating substantial representation of previously reported disease-associated genes within the cohort.

A total of 10,220 molecular variants were identified within these genes, showing a heterogeneous genomic distribution and an average sequencing depth of approximately 100×. Following filtering, only 1187 synonymous variants were excluded because of their limited predicted functional impact, resulting in 9033 variants retained for downstream analysis. Importantly, this remaining dataset still contained coding and non-coding variants, including intronic variants, untranslated region (UTR) variants, regulatory variants, insertions/deletions (INDELs), start/stop codon variants, and other uncharacterized genomic variants.

Among the retained variants, 955 corresponded to non-synonymous SNVs, of which twenty-six had been previously reported in association with Glaucoma according to DisGeNET, whereas 929 represented potentially novel coding variants requiring further investigation. The remaining 8078 variants consisted of non-SNV molecular variants, including 36 INDELs, ten start/stop codon variants, and 8032 intronic, UTR, regulatory, or other non-coding variants (Figure 1).

### 3.3. Glaucoma-Associated Variants

The analysis identified several SNPs previously associated with glaucoma according to DisGeNET (Table A1). These variants were in key genes involved in ocular development, extracellular matrix (ECM) remodeling, vascular regulation, and oxidative stress (Table 2).

Notably, variants in *SIX6* (rs33912345) and *LOXL1* (rs1048661, rs3825942) were consistently detected, supporting their well-established role in glaucoma susceptibility. Additionally, variants in *CYP1B1*, *NOS3*, and *SOD2* were identified, further reinforcing the contribution of metabolic, vascular, and oxidative pathways.

Overall, the presence of these SNPs across the cohort highlights the enrichment of previously validated glaucoma-associated loci in the analyzed population.

### 3.4. Rare Candidate Variants

Two rare variants (MAF < 1%) were identified in a high proportion of individuals within the cohort (Table 3), suggesting potential candidate variants requiring further validation for glaucoma susceptibility.

The variant located in *FMNL2* (chr2:152619552) corresponds to a coding frameshift alteration, indicating a possible functional impact on cytoskeletal dynamics. In contrast, the variant identified in *C3* (chr19:6710561) is intronic but may influence gene regulation, particularly given the known role of complement activation in neuroinflammation.

Their recurrence may reflect population-specific patterns or shared genetic background, either a shared genetic background or population-specific enrichment, warranting further investigation. Allele frequencies retrieved from gnomAD indicated that both variants are rare across all major ancestral populations, supporting their classification as rare candidate variants; however, additional studies in larger Colombian and Latin American cohorts are required to determine whether they represent population-specific enrichment.

### 3.5. Pharmacogenomic Variants

The pharmacogenomic analysis revealed variants associated with differential response to glaucoma treatments (Table 4).

Variants in *PTGS1* (rs3842787) have been previously associated with increased response to latanoprost, while *ADRB1* (rs1801252) variants were linked to potential adverse responses to timolol. Additionally, variants in *ABCC4* (rs11568658) were associated with improved drug efficacy.

These findings suggest that genetic variability may contribute to differences in therapeutic response, supporting the relevance of pharmacogenomics in glaucoma management.

### 3.6. Functional Pathway Integration

The integration of SNP-associated genes into biology suggested a functional grouping (Table 5).

Most variants were associated with:

- Oxidative stress and DNA damage
- Extracellular matrix remodeling
- Vascular regulation
- Neurodegeneration
- Inflammatory response

This distribution is consistent with the concept that glaucoma results from the interaction of multiple pathogenic mechanisms rather than a single molecular pathway.

## 4. Discussion

This study provides an exploratory characterization of the genetic landscape of primary POAG in a Colombian cohort, leveraging a targeted exome-based approach anchored in genes with prior evidence of disease association. By prioritizing variants within a curated set of glaucoma-associated genes derived from DisGeNET, the analysis was designed to enhance biological interpretability while acknowledging the constraints imposed by a limited sample size and the absence of a control group.

A key aspect of the study design was the gene prioritization strategy, which focused on a predefined list of 446 genes previously associated with POAG. The detection of 381 of these genes in the patients’ exomes indicates a substantial representation of known disease-associated loci within the cohort. This targeted approach allowed the analysis to be grounded in established biological knowledge, thereby reducing the dimensionality of the search space and mitigating the risk of spurious findings inherent to unrestricted genome-wide analyses in small cohorts.

Within this framework, the identification of previously reported glaucoma-associated variants, particularly in *SIX6*, *LOXL1*, and *CYP1B1*, confirms the presence of canonical genetic risk factors within this population. Variants in *SIX6* have been consistently associated with retinal nerve fiber layer thinning and increased susceptibility to POAG, through their role in retinal ganglion cell (RGC) development and survival [18]. Likewise, *LOXL1* variants are strongly linked to pseudo exfoliation glaucoma, where they influence extracellular matrix (ECM) organization and elastin cross-linking, thereby altering trabecular meshwork biomechanics and aqueous humor outflow [19]. These findings are consistent with large-scale genome-wide association studies (GWAS), which have demonstrated that genes involved in ocular development and structural integrity play a significant role in glaucoma susceptibility [20].

Beyond previously characterized loci, this study identified rare variants in *FMNL2* and *C3* that were observed in multiple individuals, suggesting potential candidate variants that may contribute to glaucoma pathogenesis. The *FMNL2* gene encodes a formin-related protein involved in actin cytoskeleton remodeling, a process essential for maintaining trabecular meshwork contractility and regulating aqueous humor outflow [6]. Disruption of cytoskeletal dynamics has been shown to increase outflow resistance, thereby contributing to elevated IOP. In parallel, the identification of a variant in *C3* highlights the role of complement-mediated neuroinflammation. Increasing evidence indicates that activation of the complement cascade contributes to retinal ganglion cell degeneration and optic nerve damage in glaucoma [21]. These findings suggest that structural and immune-mediated mechanisms may act synergistically in disease progression.

The interpretation of rare variants should also consider population-specific allele frequencies. Colombia represents one of the most genetically admixed populations in Latin America, with varying proportions of Native American, European, and African ancestry [22]. Consequently, allele frequencies observed in this cohort may differ from those reported in global reference databases such as gnomAD [23]. Although allele frequencies across available ancestral populations were consulted to contextualize the identified variants, the small sample size and the absence of ancestry inference preclude definitive conclusions regarding population-specific enrichment. Future studies incorporating larger multi-ethnic cohorts together with ancestry-informed analyses will be essential to validate these candidate variants and determine whether they represent true population-specific susceptibility factors or rare variants shared across different populations.

The pharmacogenomic analysis further revealed variants in *PTGS1*, *ADRB1*, and *ABCC4*, which have been previously reported to influence treatment response to first-line therapies such as latanoprost and timolol. Variants in *PTGS1* are associated with prostaglandin signaling pathways that modulate intraocular pressure reduction, whereas polymorphisms in *ADRB1* can alter beta-adrenergic receptor function and influence the efficacy of beta-blockers [24,25]. Additionally, *ABCC4* encodes a transporter involved in drug efflux, which may affect ocular drug bioavailability and therapeutic response [26]. These findings support the growing relevance of pharmacogenomics in glaucoma management and highlight the potential for personalized therapeutic strategies.

From a biology perspective, the pathway-based classification of variants provides a coherent framework linking genetic alterations to disease mechanisms. Variants associated with oxidative stress, including *SOD2*, *OGG1*, *GSTP1*, and *MTHFR*, suggest increased susceptibility to mitochondrial dysfunction and reactive oxygen species (ROS) accumulation, which are well-established contributors to retinal ganglion cell degeneration [27]. Concurrently, alterations in ECM-related genes such as *LOXL1*, *MMP9*, and *COL11A1* may disrupt trabecular meshwork architecture, leading to impaired aqueous humor outflow and increased IOP [28].

Vascular dysregulation also emerges as a critical component of glaucoma pathophysiology. Variants in *NOS3* and *EDN1* affect nitric oxide signaling and endothelin-mediated vasoconstriction, respectively, which can impair ocular blood flow and contribute to optic nerve ischemia [29]. This supports the vascular hypothesis of glaucoma, particularly in cases where disease progression occurs independently of elevated IOP.

Neurodegeneration is further supported by variants in genes such as *BDNF* and *TP53*, which regulate neuronal survival and apoptosis. Reduced neurotrophic support and increased susceptibility to programmed cell death have been identified as key drivers of retinal ganglion cell loss in glaucoma [30]. In addition, inflammatory pathways involving *TLR4* and *C3* underscore the role of innate immunity in disease progression, as chronic low-grade neuroinflammation has been shown to exacerbate neuronal damage [31].

Taken together, these findings are consistent with an integrative and system-level model of glaucoma in which oxidative stress, extracellular matrix remodeling, vascular dysfunction, neurodegeneration, and inflammation interact dynamically to drive disease onset and progression. This framework aligns with contemporary paradigms that conceptualize glaucoma as a multifactorial neurodegenerative disease involving both local ocular and systemic mechanisms [32].

Despite these advances, several limitations should be considered when interpreting the present findings. First, the small sample size (n = 21) limits the statistical power of the study and reduces the ability to detect less frequent variants or establish robust genotype–phenotype associations. Consequently, the reported frequencies should be interpreted as exploratory observations rather than definitive estimates of variant prevalence in the Colombian population.

Second, the absence of a healthy control group prevents direct comparisons of allele frequencies between affected and unaffected individuals, precluding the calculation of association measures such as odds ratios, confidence intervals, and statistical significance. Therefore, although several variants previously associated with POAG were identified and biologically plausible candidate variants were detected, the present study cannot determine whether these variants are specifically enriched in Colombian patients with glaucoma or simply reflect background population genetic variation.

In addition, the Colombian population is characterized by substantial genetic admixture, including Native American, European, and African ancestral components. Without an appropriate control cohort matched for ancestry, it is not possible to completely exclude the potential influence of population stratification on the observed distribution of genetic variants. Consequently, the findings should be interpreted primarily as hypothesis-generating rather than confirmatory.

Despite these limitations, the study provides one of the first exploratory whole-exome analyses of primary open-angle glaucoma in a Colombian cohort and identifies biologically relevant variants and pathways that warrant further investigation. Future studies should include larger, well-characterized case–control cohorts representing the genetic diversity of the Colombian population, together with functional validation of candidate variants and replication in independent populations. Such approaches will be essential to confirm the biological relevance, estimate the magnitude of genetic associations, and evaluate the clinical utility of these variants for glaucoma risk prediction and precision medicine.

## 5. Conclusions

Our findings suggest that patients with POAG harbor genetic variants across multiple interconnected biological pathways, reinforcing the multifactorial nature of the disease. The identification of both known and novel variants, particularly in *FMNL2* and *C3*, provides preliminary insights into potential mechanisms involving cytoskeletal regulation and neuroinflammation.

Furthermore, the detection of pharmacogenomic variants associated with treatment response highlights the potential relevance of genomic profiling to inform therapeutic decisions. Although the findings are exploratory, they provide a solid foundation for future research integrating genomic, clinical, and functional data.

This study contributes to the growing evidence supporting precision medicine approaches in glaucoma, particularly in underrepresented populations such as those from Latin America.

These findings should be considered exploratory and provide a foundation for future statistically powered case–control studies aimed at validating the reported genetic associations.

## Figures and Tables

**Table 1:** Demographics and clinical characteristics.

Variable	Total (n = 21)
Age (years), mean ± SD	67 ± 12.2
Age range	44–87
Female, n (%)	11 (52%)
Male, n (%)	10 (48%)
Baseline IOP (mmHg)	25.087 +/− 3.82
Treated IOP (mmHg)	17.57 +/− 4.0035
Latanoprost monotherapy, n (%)	20 (95%)
Timolol monotherapy, n (%)	1 (5%)
Bilateral glaucoma, n (%)	19 (90.5%)
Unilateral glaucoma, n (%)	2 (9.5%)

**Table 2:** SNPs previously associated with glaucoma identified in the study cohort based on DisGeNET annotation.

SNP ID	Gene	Variant Type	Functional Role	Reported Association
rs33912345	*SIX6*	Missense	Ocular development	POAG (GWAS)
rs1048661	*LOXL1*	Missense	ECM remodeling	Pseudo exfoliation glaucoma
rs3825942	*LOXL1*	Missense	ECM remodeling	Pseudo exfoliation glaucoma
rs1056836	*CYP1B1*	Missense	Xenobiotic metabolism	Glaucoma
rs1799983	*NOS3*	Missense	Nitric oxide signaling	Vascular dysfunction
rs4880	*SOD2*	Missense	Antioxidant defense	Oxidative stress

**Table 3:** Rare variants (MAF < 1%) identified with a high proportion of individuals, representing potential novel candidates associated with glaucoma.

Genomic Position	Gene	Variant	MAF (Reference Population)	Functional Prediction
chr2:152619552	*FMNL2*	−|CCA	<0.001	Coding/frameshift
chr19:6710561	*C3*	−|AA	<0.001	intronic

**Table 4:** Pharmacogenomic variants identified in the cohort with potential implications for treatment response to latanoprost and timolol.

Chromosome	SNP	Gene	Drug	Clinical Effect	Allele Frequency
chr9	rs3842787	*PTGS1*	Latanoprost	Increased response	0.095
chr10	rs1801252	*ADRB1*	Timolol	Adverse response	0.238
chr13	rs11568658	*ABCC4*	Latanoprost	Improved efficacy	0.023

**Table 5:** Classification of identified SNP-associated genes according to major biological pathways involved in glaucoma pathophysiology.

Biological Pathway	Genes	Functional Implication
Oxidative stress	*SOD2*, *OGG1*, *GSTP1*, *MTHFR*	ROS accumulation and DNA damage
ECM remodeling	*LOXL1, MMP9, COL11A1*	Trabecular meshwork dysfunction
Vascular regulation	*NOS3, EDN1*	Impaired ocular blood flow
Neurodegeneration	*BDNF, TP53*	Retinal ganglion cell loss
Inflammation	*TLR4, PTGS1*	Neuroinflammatory response

## Data Availability

The original contributions presented in this study are included in the article. Further inquiries can be directed at the corresponding author.

## Appendix A

All molecular variants with prior association to primary open-angle glaucoma identified with DisGeNET information are documented in Table A1.

**Table A1:** Molecular variants previously associated with primary open-angle glaucoma (POAG) were identified in the patients.

Chr	Position	Ref	Alt	Gen	rsID	HTC	HMC	Total	Vda Score
chr1	102914362	G	A	*COL11A1*	rs3753841	9	0	9	0.7
chr9	22012423	G	A	*CDKN2B-AS1*	rs545226	7	14	21	0.7
chr9	22029446	G	A	*CDKN2B-AS1*	rs10965215	11	4	15	0.7
chr9	22029548	T	C	*CDKN2B-AS1*	rs564398	3	0	3	0.7
chr14	60509819	C	A	*SIX6*	rs33912345	7	1	8	0.07
chr15	73927241	G	A	*LOXL1*	rs3825942	6	4	10	0.07
chr15	73927205	G	T	*LOXL1*	rs1048661	8	1	9	0.06
chr10	13110400	T	A	*OPTN*	rs11258194	4	0	4	0.05
chr17	7676154	G	C	*TP53*	rs1042522	11	6	17	0.04
chr1	171652385	C	T	*MYOC*	rs2234926	5	0	5	0.03
chr9	117713024	A	G	*TLR4*	rs4986790	1	0	1	0.03
chr19	43551574	T	C	*XRCC1*	rs25487	5	16	21	0.03
chr1	45331833	C	G	*MUTYH*	rs3219489	8	0	8	0.02
chr2	38075034	C	A	*CYP1B1*	rs1056827	11	2	13	0.02
chr7	150999023	T	G	*NOS3*	rs1799983	3	17	20	0.02
chr14	99691630	A	G	*CYP46A1*	rs754203	4	0	4	0.02
chr20	46011586	A	G	*MMP9*	rs17576	11	3	14	0.02
chr1	11796321	G	A	*MTHFR*	rs1801133	6	1	7	0.01
chr1	36099217	C	T	*COL8A2*	rs75864656	2	0	2	0.01
chr2	38070996	T	C	*CYP1B1*	rs1800440	1	0	1	0.01
chr2	38071060	G	C	*CYP1B1*	rs1056836	9	10	19	0.01
chr2	38075247	G	C	*CYP1B1*	rs10012	11	4	15	0.01
chr3	9757089	C	G	*OGG1*	rs1052133	8	0	8	0.01
chr3	123150194	C	T	*PDIA5*	rs11720822	3	1	4	0.01
chr3	193637285	T	C	*OPA1*	rs166850	0	21	21	0.01
chr3	193637313	T	C	*OPA1*	rs10451941	12	1	13	0.01
chr4	147542688	G	C	*EDNRA*	rs5335	7	12	19	0.01
chr5	111100751	C	T	*WDR36*	rs10038177	8	12	20	0.01
chr5	111103810	A	G	*WDR36*	rs11241095	5	1	6	0.01
chr5	148826910	G	C	*ADRB2*	rs1042714	5	16	21	0.01
chr6	159692840	A	G	*SOD2*	rs4880	9	3	12	0.01
chr7	151012483	G	T	*NOS3*	rs7830	8	4	12	0.01
chr10	13124076	G	A	*OPTN*	rs386741044	1	0	1	0.01
chr11	27658369	C	T	*BDNF*	rs6265	2	0	2	0.01
chr11	34438925	A	T	*CAT*	rs7943316	8	5	13	0.01
chr11	67585218	A	G	*GSTP1*	rs1695	12	4	16	0.01
chr13	95210754	C	A	*ABCC4*	rs11568658	1	0	1	0.01

Note: Chr: Chromosome; Ref: Reference allele; Alt: Alternate allele; HTC: Total number of heterozygous individuals for the variant; HMC: Total number of homozygous individuals for the variant; Vda score: Variant–disease association score (DisGeNET).
